# Delineating network integration and segregation in the pathophysiology of functional neurological disorder

**DOI:** 10.1093/braincomms/fcaf195

**Published:** 2025-05-21

**Authors:** Christiana Westlin, Andrew J Guthrie, Cristina Bleier, Sara A Finkelstein, Julie Maggio, Jessica Ranford, Julie MacLean, Ellen Godena, Daniel Millstein, Sara Paredes-Echeverri, Jennifer Freeburn, Caitlin Adams, Christopher D Stephen, Ibai Diez, David L Perez

**Affiliations:** Functional Neurological Disorder Research Group, Department of Neurology, Massachusetts General Hospital, Mass General Brigham, Harvard Medical School, Boston, MA 02114, USA; Athinoula A. Martinos Center for Biomedical Imaging, Massachusetts General Hospital, Mass General Brigham, Harvard Medical School, Boston, MA 02129, USA; Department of Psychiatry, Massachusetts General Hospital, Mass General Brigham, Harvard Medical School, Boston, MA 02114, USA; Functional Neurological Disorder Research Group, Department of Neurology, Massachusetts General Hospital, Mass General Brigham, Harvard Medical School, Boston, MA 02114, USA; Athinoula A. Martinos Center for Biomedical Imaging, Massachusetts General Hospital, Mass General Brigham, Harvard Medical School, Boston, MA 02129, USA; Functional Neurological Disorder Research Group, Department of Neurology, Massachusetts General Hospital, Mass General Brigham, Harvard Medical School, Boston, MA 02114, USA; Athinoula A. Martinos Center for Biomedical Imaging, Massachusetts General Hospital, Mass General Brigham, Harvard Medical School, Boston, MA 02129, USA; Functional Neurological Disorder Research Group, Department of Neurology, Massachusetts General Hospital, Mass General Brigham, Harvard Medical School, Boston, MA 02114, USA; Functional Neurological Disorder Research Group, Department of Neurology, Massachusetts General Hospital, Mass General Brigham, Harvard Medical School, Boston, MA 02114, USA; Department of Physical Therapy, Massachusetts General Hospital, Mass General Brigham, Boston, MA 02114, USA; Functional Neurological Disorder Research Group, Department of Neurology, Massachusetts General Hospital, Mass General Brigham, Harvard Medical School, Boston, MA 02114, USA; Department of Occupational Therapy, Massachusetts General Hospital, Mass General Brigham, Boston, MA 02114, USA; Functional Neurological Disorder Research Group, Department of Neurology, Massachusetts General Hospital, Mass General Brigham, Harvard Medical School, Boston, MA 02114, USA; Department of Occupational Therapy, Massachusetts General Hospital, Mass General Brigham, Boston, MA 02114, USA; Functional Neurological Disorder Research Group, Department of Neurology, Massachusetts General Hospital, Mass General Brigham, Harvard Medical School, Boston, MA 02114, USA; Functional Neurological Disorder Research Group, Department of Neurology, Massachusetts General Hospital, Mass General Brigham, Harvard Medical School, Boston, MA 02114, USA; Department of Psychiatry, Massachusetts General Hospital, Mass General Brigham, Harvard Medical School, Boston, MA 02114, USA; Functional Neurological Disorder Research Group, Department of Neurology, Massachusetts General Hospital, Mass General Brigham, Harvard Medical School, Boston, MA 02114, USA; Athinoula A. Martinos Center for Biomedical Imaging, Massachusetts General Hospital, Mass General Brigham, Harvard Medical School, Boston, MA 02129, USA; Functional Neurological Disorder Research Group, Department of Neurology, Massachusetts General Hospital, Mass General Brigham, Harvard Medical School, Boston, MA 02114, USA; Department of Speech, Language, and Swallowing Disorders, Massachusetts General Hospital, Mass General Brigham, Boston, MA 02114, USA; Functional Neurological Disorder Research Group, Department of Neurology, Massachusetts General Hospital, Mass General Brigham, Harvard Medical School, Boston, MA 02114, USA; Department of Psychiatry, Massachusetts General Hospital, Mass General Brigham, Harvard Medical School, Boston, MA 02114, USA; Functional Neurological Disorder Research Group, Department of Neurology, Massachusetts General Hospital, Mass General Brigham, Harvard Medical School, Boston, MA 02114, USA; Movement Disorders Division, Department of Neurology, Massachusetts General Hospital, Mass General Brigham, Harvard Medical School, Boston, MA 02114, USA; Functional Neurological Disorder Research Group, Department of Neurology, Massachusetts General Hospital, Mass General Brigham, Harvard Medical School, Boston, MA 02114, USA; Computational Neuroimaging Lab, Biobizkaia Health Research Institute, Barakaldo 48903, Spain; Ikerbasque, Baske Foundation for Science, Bilbao 48009, Spain; Gordon Center for Medical Imaging, Department of Radiology, Massachusetts General Hospital, Harvard Medical School, Boston, MA 02114, USA; Functional Neurological Disorder Research Group, Department of Neurology, Massachusetts General Hospital, Mass General Brigham, Harvard Medical School, Boston, MA 02114, USA; Athinoula A. Martinos Center for Biomedical Imaging, Massachusetts General Hospital, Mass General Brigham, Harvard Medical School, Boston, MA 02129, USA; Department of Psychiatry, Massachusetts General Hospital, Mass General Brigham, Harvard Medical School, Boston, MA 02114, USA

**Keywords:** functional neurological disorder, resting-state fMRI, integration, segregation, graph theory

## Abstract

Functional neurological disorder (FND) is a neuropsychiatric condition that is framed as a multi-network brain problem. Despite this conceptualization, studies have generally focused on specific regions or connectivity features, under-characterizing the complex and nuanced role of resting-state networks in FND pathophysiology. This study employed three complementary graph theory analyses to delineate the functional network architecture in FND. Specifically, we investigated whole-brain weighted-degree, isocortical integration and isocortical segregation extracted from resting-state functional MRI data prospectively collected from 178 participants: 61 individuals with mixed FND; 58 psychiatric controls matched on age, sex, depression, anxiety and post-traumatic stress disorder severity; and 59 age- and sex-matched healthy controls. All analyses were adjusted for age, sex and antidepressant use and focused on differences between FND versus psychiatric controls, with individual-subject maps normalized to healthy controls. Compared to psychiatric controls, patients with mixed FND exhibited increased weighted-degree in the right dorsal anterior cingulate and superior frontal gyrus and the left inferior frontal gyrus and supplementary motor area. Isocortical integration analyses revealed increased *between-network* connectivity for somatomotor network areas, with widespread heightened connections to regions of the default mode, frontoparietal and salience networks. Isocortical segregation analyses revealed increased *within-network* connectivity for the frontoparietal network. Secondary analyses of functional motor disorder (*n* = 46) and functional seizure (*n* = 23) subtypes (versus psychiatric controls) revealed both shared and unique patterns of altered connectivity across subtypes, including increased weighted-degree and integrated connectivity in the left posterior insula and anterior/mid-cingulate in functional motor disorder and increased segregated connectivity in the right angular gyrus for functional seizures. In *post hoc* between-group analyses, findings remained significant adjusting for depression, anxiety and post-traumatic stress disorder severity, as well as for childhood maltreatment. *Post hoc* correlations revealed significant relationships between connectivity metrics in several of these regions and somatic symptom severity across FND and psychiatric control participants. Notably, individual connectivity values were predominantly within the range of healthy controls (with patients with FND generally showing tendencies for increased connectivity and psychiatric controls showing tendencies towards decreased connectivity), indicating subtle shifts in the network architecture rather than gross abnormalities. This study provides novel mechanistic insights (i.e. increased somatomotor integration) and specificity regarding the neurobiology of FND, highlighting both shared mechanisms across subtypes and subtype-specific patterns. The results support the notion that FND involves aberrant *within*- and *between-network* communication, setting the stage for biologically informed treatment development and large-scale replication.

## Teaser

Westlin *et al*. report that patients with functional neurological disorder, compared to psychiatric controls, had increased between-network integration for somatomotor network areas and increased within-network segregation for the frontoparietal network—findings that correlated with somatic symptom severity. Results support the notion that the disorder involves aberrant within- and between-network communication.

## Graphical Abstract

**Graphical Abstract:**
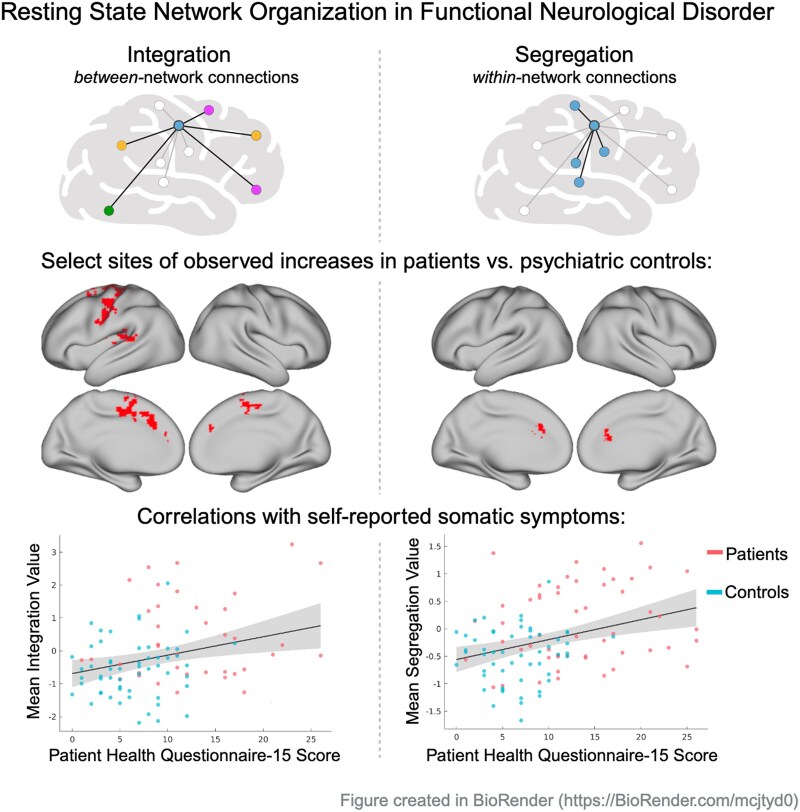

## Introduction

Functional neurological disorder (FND) is a neuropsychiatric condition that presents with distressing sensorimotor and cognitive symptoms.^1-3^ While the most well-characterized FND subtypes are functional motor disorder (FND-motor) and functional seizures (FND-seiz^4^), patients frequently exhibit mixed symptoms and develop new FND symptoms longitudinally.^5-7^ Other physical symptoms (e.g. pain, fatigue), psychiatric comorbidities and adverse life experiences are also present in many with FND.^3,5,8-12^ This heterogeneity presents a challenge for understanding mechanisms of the disorder, necessitating a multifaceted approach that combines insights across subtypes and contextualizes findings alongside psychiatric comorbidities.^13,14^

Neuroimaging advances have provided insights into FND pathophysiology, identifying altered brain function and structure across regions of the somatomotor, salience and default mode networks, among other findings.^14,15^ One commonly used method, resting-state functional MRI (rsfMRI), can reveal altered functional connectivity relationships in FND. Specifically, seed-based analyses have delineated aberrant connectivity between the salience network and motor control regions, as well as between the temporoparietal junction and sensorimotor areas.^16-21^ Other studies have used independent component analysis,^22,23^ dynamic connectivity investigations^24-26^ and machine learning to characterize the functional architecture of FND.^27,28^ Nonetheless, studies have generally focused on specific regions or connectivity features, under-characterizing the complex and integrative nature of brain networks.

A more comprehensive understanding of FND can come from examining the functional network architecture using graph theory.^29^ Brain networks exhibit ‘small-world’ organization, with densely interconnected areas (i.e. modules or communities), as well as cross-modular connections that enable efficient information flow between communities.^30,31^ Modelling the brain as a mathematical ‘graph’ with nodes (regions) and edges (region-to-region relationships) provides a powerful tool to probe network connections. Several FND studies have used graph theory to identify altered somatomotor, salience and default mode network properties.^32-35^ However, these and previously discussed studies have used only healthy controls (HCs), often also focusing on one FND subtype. Our research group previously compared brain-childhood maltreatment relationships in a separate FND cohort versus psychiatric controls (PCs) using graph theory; however, no quantitative between-group comparisons were performed and the investigation was limited to the differential impact of childhood abuse and neglect.^33^ As such, it remains unclear whether the emerging network architecture of FND is related to co-occurring psychiatric conditions (and shared risk factors), associated with common processes across FND subtypes or linked to specific FND presentations.

Here, we employed three graph theory analyses to characterize the functional network architecture in a mixed FND cohort (FND-mixed) compared to PCs and HCs. Specifically, we investigated whole-brain weighted-degree, isocortical integration and isocortical segregation. Weighted-degree measures centrality, quantifying the influence of a given region within the whole-brain architecture.^36^ Integration refers to the ability to combine information across distributed functional networks, indexing *between-network* communication.^30,36-39^ Segregation refers to the ability to process information *within* a discrete network.^29,37,39^ Examining integration and segregation, concepts not previously studied in FND, has led to important mechanistic insights in other neuropsychiatric disorders.^40-42^ These complementary measures help delineate aberrant communication *within* a given network and/or *across* two or more brain networks in FND. Furthermore, subtype-specific effects were evaluated by investigating functional connectivity in FND-motor and FND-seiz versus PCs separately. We also performed *post hoc* corrections to account for affective symptoms and childhood maltreatment burden. Additionally, we investigated how differences in network architecture related to the severity of core FND symptoms as well as non-core somatic symptoms.^43^ We aimed to delineate neurobiological characteristics shared across FND-mixed, as well as potential subtype differences, offering insights into mechanisms and potential biologically informed diagnostic and therapeutic targets.

## Methods

### Participants

Sixty-one FND-mixed participants [52 female; mean age (SD) = 40.1 ± 13.9 years; average illness duration = 4.2 ± 5.4 years, range = 0.3–25 years; Table 1] were prospectively recruited from the Massachusetts General Hospital between June 2018 and March 2024.^4,44^ Diagnoses were made using positive signs, semiological features and electroencephalography data (FND-seiz only^45^). Exclusion criteria were major neurological comorbidities (e.g. epilepsy, Parkinson’s disease), known brain MRI abnormalities, poorly controlled medical problems with central nervous system (CNS) consequences, active illicit substance dependence, known psychosis and/or active suicidality. The FND-mixed cohort included 46 FND-motor (tremor = 20; weakness = 17; gait = 16; speech = 14; tics/jerks/spasms = 9; dystonia = 3) and 23 FND-seiz (documented = 17; clinically established = 2; probable = 4) subjects (Supplementary Table 1). Eight participants had both FND-motor and FND-seiz. We employed a transdiagnostic approach across FND-motor and FND-seiz, given clinical and mechanistic overlap,^46^ while systematically considering subtype effects (Supplementary Table 2). Data from this cohort were previously published in a structural MRI study^47^; however, rsfMRI data have not been previously published.

**Table 1:** Demographic and psychometric characteristics of FND-mixed, PC and HC samples

	FND-mixed (*N* = 61) Mean ± SD or *N*	PCs (*N* = 58) Mean ± SD or *N* (*P* corrected)	HCs (*N* = 59) Mean ± SD or *N* (*P* corrected)
Age (years)	40.1 ± 13.9	35.7 ± 13.3 (0.2)	36.6 ± 10.9 (0.3)
Sex	F: 52; M: 9	F: 49; M: 9 (0.9)	F: 48; M: 11 (0.6)
SDQ-20	34.7 ± 12.4	22.0 ± 4.5 (<0.001*)	20.3 ± 0.6 (<0.001*)
PHQ-15	13.2 ± 6.2	6.3 ± 3.7 (<0.001*)	2.6 ± 2.2 (<0.001*)
BDI-II	16.8 ± 12.0	14.4 ± 13.2 (0.3)	1.5 ± 2.5 (<0.001*)
STAI-Total	82.5 ± 22.0	79.1 ± 23.7 (0.5)	53.9 ± 9.3 (<0.001*)
PCL-5	27.6 ± 19.0	22.7 ± 18.9 (0.3)	2.7 ± 3.6 (<0.001*)
CTQ-Abuse	32.3 ± 13.0	26.5 ± 12.2 (0.03*)	17.9 ± 3.4 (<0.001*)
CTQ-Neglect	21.0 ± 8.7	19.8 ± 8.9 (0.5)	13.2 ± 4.3 (<0.001*)
SSRI/SNRI	32	27 (0.6)	0 (<0.001*)

*P* values reflect statistical significance between the FND cohort and the control group after FDR correction for multiple comparisons. Asterisks indicate corrected *P* < 0.05. Statistical comparisons of continuous variables were done using a Mann–Whitney U-test, while comparisons of discrete variables were done using a *χ*² test. Five FND and one PC participants had incomplete data. For each variable, counts of missing data were as follows: SDQ-20: five FND, one PC; PHQ-15: five FND; BDI-II: four FND; STAI-total: four FND; PCL-5: five FND; CTQ: four FND.

F, female; M, male; SDQ-20, Somatoform Dissociation Questionnaire-20; PHQ-15, Patient Health Questionnaire-15; BDI-II, Beck Depression Inventory-II; STAI-Total, Spielberger State-Trait Anxiety Inventory-Total; PCL-5, Post-Traumatic Stress Disorder Checklist for DSM-5; CTQ, Childhood Trauma Questionnaire; SSRI/SNRI, selective serotonin reuptake inhibitor/serotonin norepinephrine reuptake inhibitor use.

Fifty-eight PCs [49 female; mean age (SD) = 35.7 ± 13.3 years] were prospectively recruited from the community. PCs had a history of clinically salient depression (*n* = 50), anxiety (*n* = 42) and/or post-traumatic stress disorder (PTSD; *n* = 20). Forty-one participants had a history of more than one of these psychiatric diagnoses. Exclusion criteria were the same as for FND, with the added exclusion of a FND or somatic symptom disorder diagnosis (Supplementary Table 3).

Fifty-nine HCs [48 female; mean age (SD) = 36.6 ± 10.9 years] were prospectively recruited from the community. HCs had no psychiatric, major neurological or poorly controlled medical conditions with known CNS consequences. No HCs were on psychotropic medications.

Twenty-six additional subjects (7 FND, 10 PCs, 9 HCs) were enrolled but excluded following imaging acquisition and processing. Seventeen participants (four FND, six PCs, seven HCs) were excluded due to excessive head motion (<120 usable volumes), and nine participants (three FND, four PCs, two HCs) were excluded from all analyses following outlier inspection. Outliers were defined as having a mean weighted-degree at the whole-brain level exceeding more than 1.5 times the interquartile range above the upper quartile or below the lower quartile. All subjects signed informed consent, and the Mass General Brigham Institutional Review Board approved this study.

### Neuropsychiatric characterization

All participants completed a Structured Clinical Interview for Diagnostic and Statistical Manual Disorders (SCID-I). FND severity was measured using the Somatoform Dissociation Questionnaire-20 (SDQ-20): a 20-item scale evaluating the extent to which core FND symptoms (e.g. paralysis) were experienced over the past year on a 5-point Likert scale.^48^ Somatic symptom severity was measured using the Patient Health Questionnaire-15 (PHQ-15): a 15-item scale measuring how bothersome physical symptoms (e.g. pain, fatigue) were over the past 4 weeks on a 3-point Likert scale.^49^ Participants also completed the Beck Depression Inventory-II (BDI-II), Spielberger State-Trait Anxiety Inventory (STAI), PTSD Checklist-5 (PCL-5) and Childhood Trauma Questionnaire (CTQ). Six participants had incomplete psychometric data (five FND, one PC).

### MRI acquisition and preprocessing

3T MRI acquisition details are described in the Supplementary Materials. Anatomical and functional MRI data were preprocessed using FMRIB Software Library v5.0.7 (FSL, Oxford, UK) and MATLAB 2023a (MathWorks, Natick, MA). The preprocessing pipelines have been described previously.^33^ Anatomical T1 preprocessing included reorientation to right-posterior-inferior (RPI); alignment to anterior and posterior commissures; skull stripping; segmentation of grey matter, white matter and cerebrospinal fluid; and computation of non-linear transformation between individual skull-stripped T1 and 3 mm resolution MNI152 template images. fMRI preprocessing included removal of the first four time points to reach a steady state; slice time correction; reorientation to RPI; realignment of functional volumes within runs via a rigid body transformation (6-parameter linear transformation); computation of the transformation between individual skull-stripped T1 images and mean functional images; intensity normalization; and removal of confounding factors using linear regression, including 12 motion-related covariates (rigid motion parameters and its derivatives), linear and quadratic terms and 5 components each from the lateral ventricles and white matter. Functional preprocessing also included transformation to MNI space, spatial smoothing with a 6 mm FWHM Isotropic Gaussian kernel and band-pass filtering (0.01–0.08 Hz). Head motion was quantified using realignment parameters, including three translation and three rotation estimates. Time points with excessive head motion (framewise displacement > 0.5 mm) were scrubbed from the data. Analyses were performed on 120 time points per subject; if participants had more than one resting-state acquisition, runs were concatenated and the first available 120 time points per subject were retained to ensure an equal number of time points were used for individual-subject connectivity matrices.

### Functional connectivity analyses

**Figure 1:**
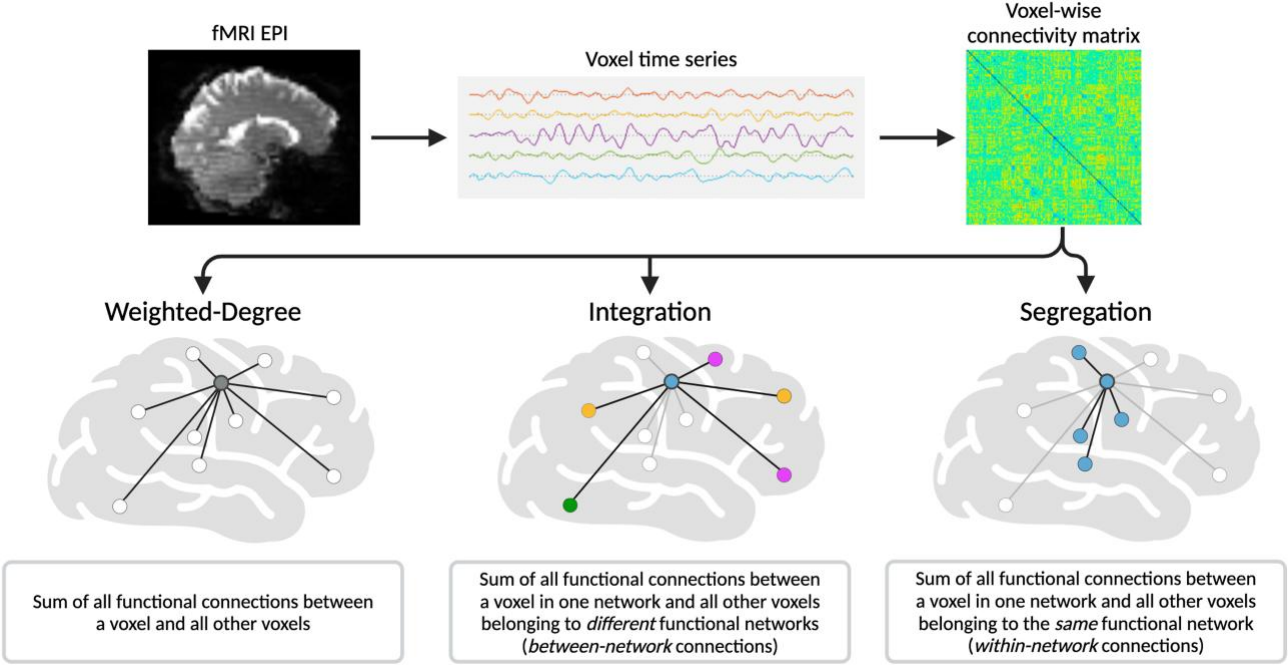
**Methodological overview of graph theory functional connectivity analyses.** A fMRI echo planar imaging acquisition for each participant was used to extract a time series for every voxel. Participant-level, voxel-wise connectivity matrices were generated by computing Pearson correlation coefficients between the time series of every pair of voxels. These connectivity matrices were then used to derive weighted-degree maps, which were computed by summing the weighted connections of a given voxel (*left*). A higher weighted-degree (centrality) value indicates that a given voxel is more functionally connected to the rest of the brain. The isocortical connections from these connectivity matrices were also used to derive integration (*middle*) and segregation (*right*) maps. If a connection started and ended in different networks, it was classified as an integration connection, whereas if the connection started and ended in the same network, it was classified as a segregation connection. Integration and segregation were then computed by summing all corresponding integration or segregation connections. A higher integration value indicates that a given voxel is an important node for across-network communication (quantifying the brain’s ability to combine information across modular communities), whereas a higher segregation value indicates that a given voxel is highly connected to other voxels within the same resting-state network (quantifying the modular organization of a given network). Created in BioRender (https://BioRender.com/wwxd8zy).

We investigated network properties via three graph theory metrics (Fig. 1). For each analysis, we first derived a resting-state functional connectivity (rsFC) matrix for each participant by computing Pearson correlation coefficients between the time series of each pair of grey matter voxels. Negative values were removed due to their controversial interpretation.^50^ Weighted-degree used whole-brain connectivity matrices, whereas integration and segregation used isocortical connections only. Each metric was computed for the FND-mixed cohort versus PCs, followed by subtype analyses for FND-motor and FND-seiz versus PCs.

#### Weighted-degree

To assess each voxel’s contribution to the overall functional architecture, we computed voxel-level weighted-degree for each participant as follows:


(1) $$WD_{i}=\;\overset{n}{\underset{\;j=1}{\sum }}\;adj(i,\;j)$$

where the weighted-degree (*WD*) for voxel *i* is computed as the sum of that voxel’s weighted connections to all other voxels *j*.

We summed the weighted connections of each voxel to generate a weighted-degree adjacency matrix per participant, which we then transformed into a map depicting the extent to which each voxel was functionally connected to the rest of the brain.

#### Integration and segregation

To examine group-level differences in isocortical integration (sum of between-network connections, quantifying the brain’s ability to combine information across communities) and segregation (sum of within-network connections, quantifying the brain’s modular organization), we first assigned each isocortical voxel to a single network based on a seven-network functional parcellation map.^29,51^ The following networks are included: visual, somatomotor (SMN), dorsal attention, ventral attention, limbic, frontoparietal (FPN) and default mode (DMN). We refer to Yeo *et al.*’s ventral attention network as the salience network (SN).^52^ If a connection’s starting and ending voxels belonged to different networks, it was classified as an integration connection, whereas if the connection started and ended in the same network, it was classified as a segregation connection.

Integration is computed as follows:


(2) $$Integration_{i}=\;\overset{n}{\underset{k=1}{\sum }}\;adj(i,k_{between-network\;voxels})$$

where the integration for voxel *i* is computed as the sum of connections to all other voxels *k* that are in a different network than *i*.

Segregation is computed as follows:


(3) $$Segregation_{i}=\;\overset{n}{\underset{l=1}{\sum }}\;adj(i,l_{within-network\;voxels})$$

where the segregation for voxel *i* is computed as the sum of connections to all other voxels *l* that are in the same network as *i*.

We summed the weighted connection of each voxel’s integration and segregation connections separately, generating subject-level integration and segregation adjacency matrices. Matrices were then transformed into brain maps depicting the extent to which each voxel was functionally integrating across networks or segregated within its own network. Higher integration values indicated that a voxel was a more important node for communicating *across* networks, whereas higher segregation values indicated that a voxel was more highly connected to other voxels *within* the same network. Given that commonly used functional parcellations only represent isocortical components, integration and segregation analyses were limited to isocortical brain areas.^51,53-56^

#### Normalization to healthy controls

Prior to statistical comparisons, we normalized the weighted-degree, integration and segregation maps for FND and PCs relative to HCs. This allows interpretation in reference to HCs, while maintaining the statistical focus on FND versus PC comparisons. We removed the effect of age and sex using a general linear model (GLM) and then normalized each participant’s map as follows:


(4) $$Normalized\;WD_{i}=\;\frac{WD_{i}-mean(WD_{HC})}{std(WD_{HC})}\;$$

where the normalized weighted-degree (*WD*) for voxel *i* was computed by subtracting the mean and dividing by the standard deviation of the HCs. The same normalization was computed for integration and segregation.

#### Statistical analysis

GLMs were used to compute FND versus PC between-group differences. In addition to the prior removal of age and sex, all GLMs also controlled for selective serotonin reuptake inhibitor/serotonin norepinephrine reuptake inhibitor (SSRI/SNRI) use (yes/no). Findings were corrected for multiple comparisons using Monte Carlo simulation clusterwise correction with 10 000 iterations to estimate the probability of false positive clusters with *P* < 0.05. *Post hoc* analyses also adjusted for (i) BDI-II, STAI-Total and PCL-5 scores and (ii) CTQ-Abuse and CTQ-Neglect scores. To examine voxels that held across all corrections, intersection maps were computed.

### Additional *post hoc* analyses

#### Seed-to-voxel connectivity analyses

We conducted two seed-based *post hoc* analyses to better understand the connectivity patterns between integration hubs and the rest of the brain. We selected one seed per hemisphere based on peak voxels in the integration map for the FND-mixed versus PC comparison and generated 3 mm spherical regions-of-interest (ROIs) around those voxels (encompassing 27 voxels centred on the peak voxel). We then computed Pearson correlations between the average time course within each seed ROI and all isocortical voxels and transformed these values using a Fisher’s r-to-z transformation. The resulting connectivity maps were then entered into a GLM to compute the group difference between FND-mixed versus PCs, adjusting for age, sex and SSRI/SNRI use. We used a Monte Carlo simulation clusterwise correction with 10 000 iterations to estimate the probability of false positive clusters with *P* < 0.05. We then applied a seven-network functional parcellation map^51^ to label each significant voxel in the connectivity map and computed the per cent of voxels in each of the six other networks (excluding the network of the seed since those connections would not be relevant for integration).

#### Symptom correlations

*Post hoc* Spearman correlations were computed between mean weighted-degree/integration/segregation values for each significant cluster and SDQ-20 and PHQ-15 scores. We computed correlations with the SDQ-20 and PHQ-15 for FND cohorts only (i.e. FND-mixed, FND-motor, FND-seiz) and correlations with only the PHQ-15 across FND and PC cohorts (FND-mixed and PCs, FND-motor and PCs, FND-seiz and PCs). Multiple comparison correction using false discovery rate corrections was done at the subtype and graph theory metric level.

## Results

### Demographic and psychometric comparisons

There were no age or sex differences between the FND-mixed, PC and HC cohorts. Compared to PCs, FND-mixed scored higher on the SDQ-20, PHQ-15 and CTQ-Abuse subscale and did not differ on BDI-II, STAI-Total, PCL-5 or CTQ-Neglect subscales; there were no differences in SSRI/SNRI use between FND and PC cohorts. Compared to HCs, FND-mixed had higher scores on all psychometric scales (see Table 1 for a complete description).

### Weighted-degree

**Figure 2:**
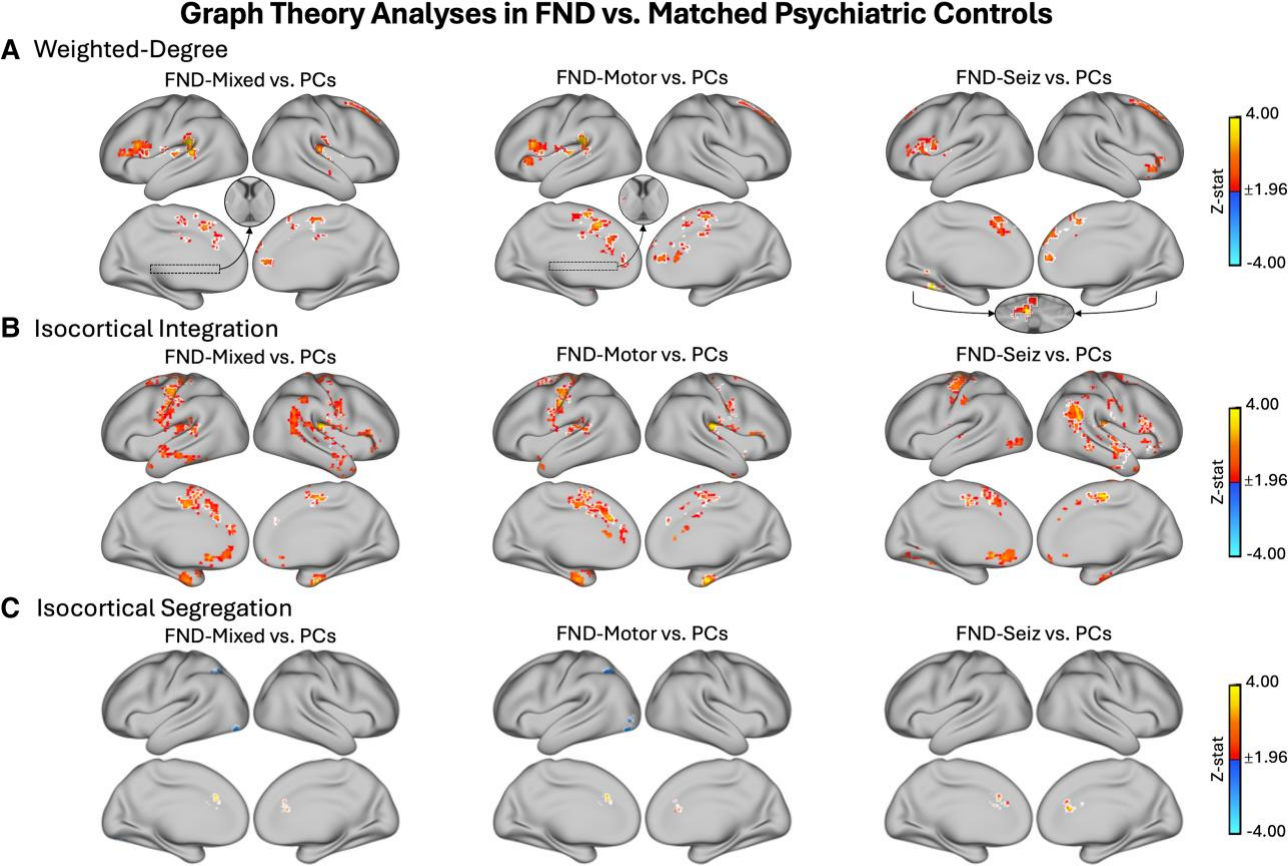
**Graph theory analyses in FND versus matched PCs.** (**A**) Results from whole-brain weighted-degree functional connectivity analyses. *Left panel* shows increased weighted-degree in patients with FND-mixed (*N*  *=* 61) compared with PCs (*N*  *=* 58) in the bilateral posterior insula and insular-opercular cortices, STG, SMG, SMA, DMPFC, ACC, MCC, right SFG and left IFG. *Middle panel* shows increased weighted-degree in FND-motor (*N*  *=* 46) compared with PCs in the bilateral ACC, MCC, SFG, SMA, DMPFC, left posterior insula and insular-opercular cortex and IFG. *Right panel* shows increased weighted-degree in patients with FND-seiz (*N*  *=* 23) compared with PCs in the bilateral SMA and SFG, right ACC, left IFG, lingual gyrus and cerebellum. (**B**) Results from isocortical integration analyses. *Left panel* shows increased integration in patients with FND-mixed compared with PCs in the bilateral posterior insula and insular-opercular cortices, ACC, MCC, postcentral gyri, SMA, DMPFC, STG, left precentral gyrus and right ITG. *Middle panel* shows increased integration for FND-motor versus PCs in largely the same areas as in FND-mixed, with the addition of right precentral gyrus and left SFG. *Right panel* shows increased integration for FND-seiz versus PCs in bilateral SMA, right posterior insula and insular-opercular cortex, IFG, angular gyrus, MTG, STG and left pre- and postcentral gyrus. (**C**) Results from isocortical segregation analyses. *Left panel* shows increased segregation in patients with FND-mixed compared with PCs in the bilateral dorsal ACC and a decreased segregation in the left IPL, lingual gyrus and inferior occipital gyrus. *Middle panel* shows a similar pattern for the FND-motor versus PC comparison. *Right panel* shows a similar pattern for increased segregation in FND-seiz versus PCs, with no regions exhibiting decreased segregation. Colours reflect the z-statistic computed from a two-sample general linear model for the primary adjustment for age, sex and SSRI/SNRI use (z-statistic > 1.96; *P* < 0.05 cluster-corrected for multiple comparisons). White outlines reflect regions that also held across all *post hoc* corrections for (i) BDI-II, STAI-Total and PCL-5 scores and (ii) CTQ-Abuse and CTQ-Neglect scores. Voxels of intersection that held across all analyses are visualized in Supplementary Fig. 1. Volumetric brain maps were visualized on the cortical surface using Connectome Workbench. FND-Mixed, mixed functional neurological disorder; FND-motor, functional motor disorder; FND-seiz, functional seizures; PCs, psychiatric controls; SSRI/SNRI, selective serotonin reuptake inhibitor/serotonin norepinephrine reuptake inhibitor; BDI-II, Beck Depression Inventory-II; STAI-Total, State Trait Anxiety Inventory-Total; PCL-5, PTSD Checklist-5; CTQ, Childhood Trauma Questionnaire; z-stat, z-statistic.

We observed increased weighted-degree for FND-mixed versus PCs in the bilateral posterior insula and insular-opercular cortices, superior temporal gyri (STG), supramarginal gyri (SMG), supplementary motor area (SMA), dorsomedial prefrontal cortex (DMPFC), anterior and mid cingulate (ACC, MCC), right superior frontal gyri (SFG) and left inferior frontal gyrus (IFG) and putamen (Fig. 2A). When focusing on FND-motor versus PCs, the FND-motor group showed a similar pattern to FND-mixed, with more widespread weighted-degree increases bilaterally in the ACC/MCC and no between-group differences in the right posterior insula and insular-opercular cortex, STG or SMG. When focusing on FND-seiz versus PCs, the FND-seiz participants had an increased weighted-degree in regions including the bilateral SMA and SFG, right ACC and left IFG, lingual gyrus and cerebellum. No regions showed decreased weighted-degree for any of the FND versus PC group comparisons. Findings held correcting *post hoc* for (i) BDI-II, STAI-Total and PCL-5 scores and (ii) CTQ-Abuse and CTQ-Neglect subscales.

Points of intersection across all weighted-degree analyses (FND-mixed versus PCs; FND-motor versus PCs; FND-seiz versus PCs) and corrections included the left IFG and opercular cortex, as well as the right ACC, SFG and the bilateral SMA (Supplementary Fig. 1A). All weighted-degree maps were normalized to HCs, such that the statistical focus was between FND versus PCs, yet results could be interpreted with reference to HCs. In relation to HCs, FND subjects had slightly higher mean weighted-degree scores in the regions of intersection, whereas PCs had slightly lower mean weighted-degree scores, but scores across both groups were predominantly in the HC range (based on visualizations; Supplementary Fig. 1A).

### Isocortical integration

We observed increased isocortical integration in the FND-mixed group versus PCs in the bilateral posterior insula and insular-opercular cortex, ACC, MCC, postcentral gyri, SMA, DMPFC, STG, left precentral gyrus and right inferior temporal gyrus (ITG). The FND-motor subtype showed increased integration compared to PCs in largely the same regions as FND-mixed, with the addition of the right precentral gyrus and left SFG. The FND-seiz subtype showed similar increased integration in the bilateral SMA, right STG, posterior insula and insular-opercular cortex and the left pre- and postcentral gyrus. FND-seiz also had increased integration in the right IFG, angular gyrus and middle temporal gyrus (MTG). No regions showed decreased integration for any of the FND versus PC group comparisons. Findings held correcting *post hoc* for (i) BDI-II, STAI-Total and PCL-5 scores and (ii) CTQ-Abuse and CTQ-Neglect subscale scores (Fig. 2B). Across all integration analyses and adjustments, points of intersection included the right posterior insula and insular-opercular cortex, ITG, bilateral SMA and left pre- and postcentral gyrus (Supplementary Fig. 1B). Mean integration values within each intersecting region were in the range of HCs, with FND-mixed participants showing slightly increased values and PCs showing slightly decreased values (Supplementary Fig. 1B).

### Integration-based seed-to-voxel connectivity

**Figure 3:**
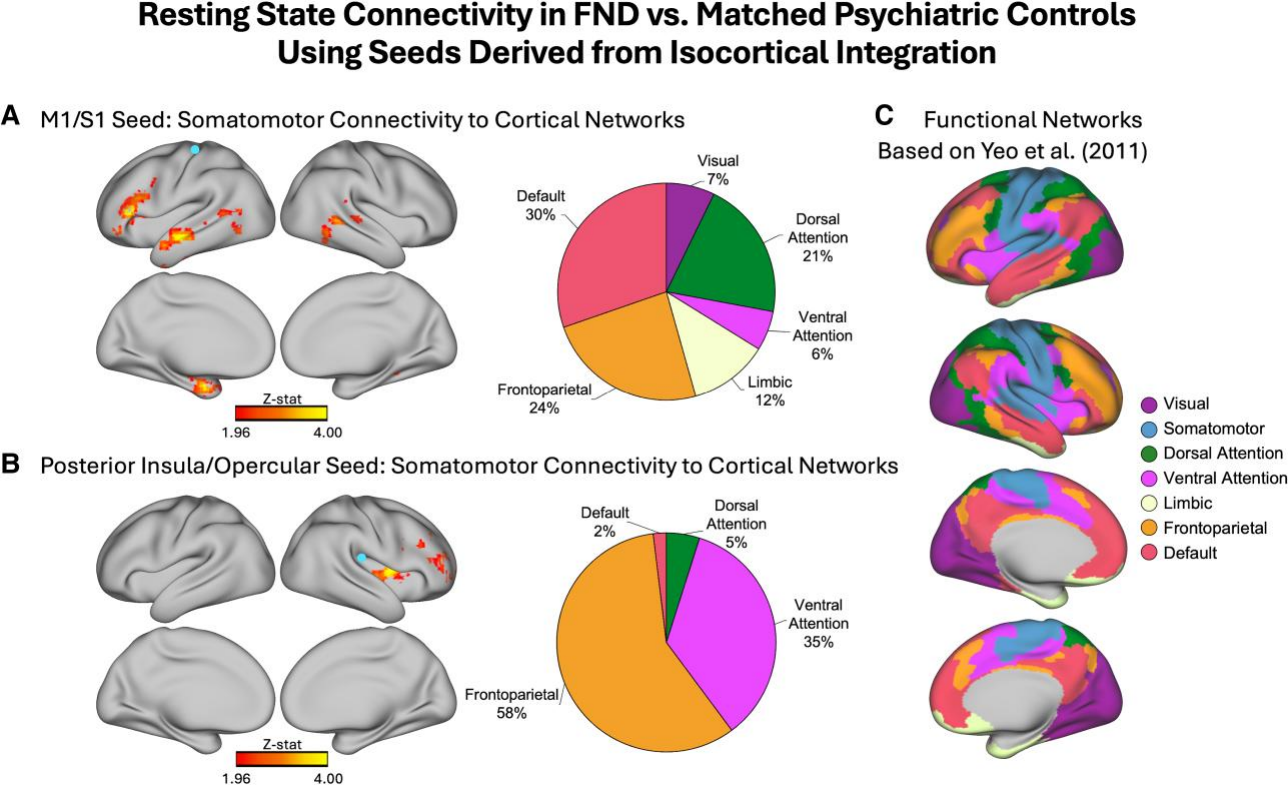
**Resting-state functional connectivity in FND versus matched PCs using seeds derived from isocortical integration.** (**A**) Seed-to-voxel connectivity in FND-mixed (*N*  *=* 61) versus PCs (*N*  *=* 58) using a seed based on the peak left hemisphere voxel in the integration analysis (indicated by the blue circle). The seed was within the somatomotor network as defined by the seven-network parcellation in Yeo *et al.*^51^ and had a pattern of increased functional connectivity in FND-mixed versus PCs with regions of all six other functional networks. Specifically, 30% of the significant voxels fell within the default mode network, 24% within the frontoparietal network, 21% within the dorsal attention network, 12% within the limbic network, 7% within the visual network and 6% within the ventral attention (salience) network. Colours reflect the z-statistic computed from a two-sample general linear model, adjusted for age, sex and SSR/SNRI use (z-stat > 1.96; *P* < 0.05 cluster-corrected for multiple comparisons). (**B**) Seed-to-voxel connectivity in FND-mixed versus PCs using a seed based on the peak right hemisphere voxel in the integration analysis (indicated by the blue circle). The seed was within the somatomotor network and had a pattern of increased functional connectivity in FND-mixed versus PCs primarily with the frontoparietal network (58% of voxels) and ventral attention (salience) network (35% of voxels), as well as the dorsal attention (5%) and default mode (2%) networks. (**C**) A visualization of the seven-network parcellation derived in Yeo *et al.*^51^ that was used to assign voxels in the connectivity maps to resting-state networks in our analyses. All volumetric brain maps were visualized on the cortical surface using Connectome Workbench. M1, primary motor cortex; S1, primary somatosensory cortex; z-stat, z-statistic.

To better understand what networks the regions showing differences in integration were functionally connected to, we conducted seed-to-voxel connectivity analyses using seeds based on regions that exhibited maximal integration differences (Fig. 3). Specifically, we generated one seed per hemisphere based on the peak voxels in the primary integration analysis for FND-mixed versus PCs that fell within the points of intersection across all analyses. The left hemisphere seed was along the pre- and postcentral gyrus, while the right hemisphere seed was within the posterior insula and insular-opercular cortex. Both seeds were within the SMN as defined by Yeo *et al.*^51^ (Fig. 3C). The left hemisphere seed had increased functional connectivity in FND-mixed versus PCs with all six other defined functional networks (DMN, FPN, SN, dorsal attention, limbic, visual; Fig. 3A). The right hemisphere seed predominantly had increased functional connectivity with the FPN and SN (Fig. 3B).

### Isocortical segregation

We observed increased isocortical segregation in FND-mixed versus PCs in the bilateral dorsal ACC and decreased segregation in the left inferior parietal lobule (IPL), lingual gyrus and inferior occipital gyrus. The same segregation findings held for the FND-motor comparison versus PCs. For FND-seiz versus PCs, there was similarly increased segregation in the bilateral dorsal ACC, but no regions exhibited decreased segregation. These findings held when correcting *post hoc* for (i) BDI-II, STAI-Total and PCL-5 scores and (ii) CTQ-Abuse and CTQ-Neglect subscale scores (Fig. 2C). For the increased bilateral ACC segregation findings across all analyses (Supplementary Fig. 1C), individuals with FND and PCs exhibited segregation values that were within the HC range, with the FND group generally having slightly higher than normal values and the PCs having slightly lower than normal values (Supplementary Fig. 1C).

### Subtype comparisons

**Figure 4:**
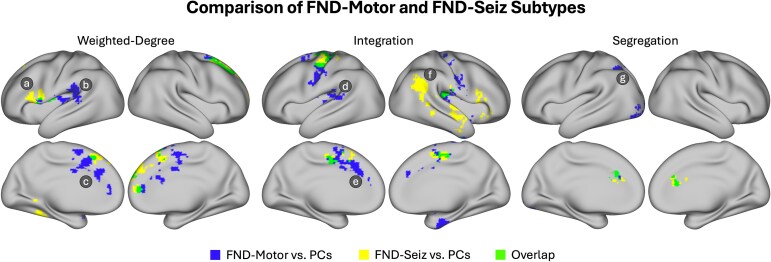
**Comparison of FND-motor and FND-seiz subtypes.** Overlays depict voxels that held across all two-sample general linear models (z-stat > 1.96; *P* < 0.05 cluster-corrected for multiple comparisons) and *post hoc* corrections for the weighted-degree (*left*), integration (*middle*) and segregation (*right*) analyses. Results for the FND-motor (*N*  *=* 46) versus PC (*N*  *=* 58) analyses are shown in blue, FND-seiz (*N*  *=* 23) versus PC analyses shown in yellow and overlapping regions across both subtype analyses shown in green. The left IFG (**A**) had increased weighted-degree that was unique to the FND-seiz group, whereas the FND-motor group had increased weighted-degree in the left posterior insula and insular-opercular cortex and left STG (**B**) and the ACC and MCC (**C**). In the integration analyses, the FND-motor group had uniquely increased integration in the left insula, insular-opercular cortex and STG (**D**), as well as the bilateral ACC and MCC (left more widespread than right) (**E**), while the FND-seiz group had a unique pattern of increased integration in the right angular gyrus, MTG and ITG (**F**). In the segregation analyses, the FND-motor subgroup had a unique pattern of decreased segregation in the left IPL (**G**), lingual and inferior occipital gyrus. All volumetric brain maps were visualized on the cortical surface using Connectome Workbench.

We next focused on brain areas that differed across FND-motor and FND-seiz subtypes (versus PCs) (Fig. 4). For weighted-degree, we observed increased weighted-degree in the left IFG that was unique to the FND-seiz group (Fig. 4A), whereas the FND-motor group had increased weighted-degree in the left posterior insula and insular-opercular cortex, as well as the left STG (Fig. 4B). The FND-motor group also had a more widespread increased weighted-degree bilaterally in the ACC and MCC (Fig. 4C).

For integration, areas with increased values unique to FND-motor subgroup included the left insula, insular-opercular cortex and STG (Fig. 4D), as well as the bilateral ACC and MCC (Fig. 4E). The right angular gyrus, MTG and ITG showed a unique pattern of increased integration in the FND-seiz subgroup (Fig. 4F). Finally, for segregation, the FND-motor subgroup showed uniquely decreased segregation in the left IPL (Fig. 4G), lingual and inferior occipital gyrus.

### Physical symptom correlations

**Figure 5:**
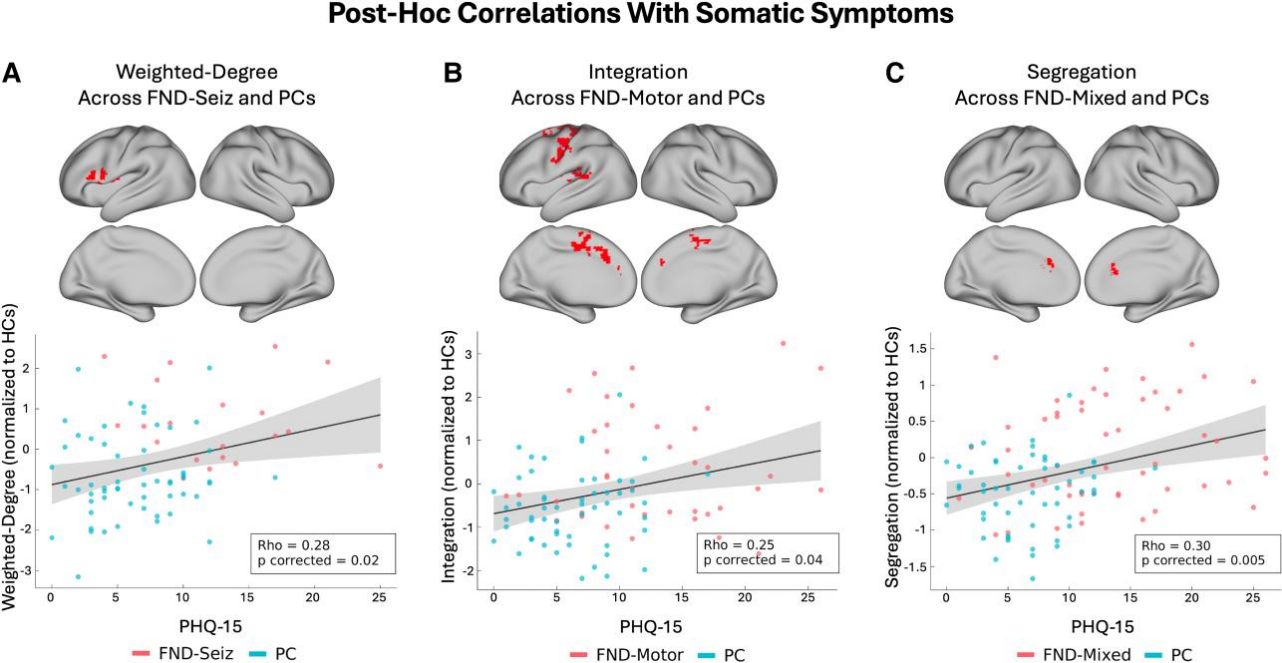
**Correlations with somatic symptom severity.** (**A**) The significant Spearman correlation across FND-seiz (*N* = 20) and PCs (*N*  *=* 56) between mean weighted-degree values extracted from the cluster shown in red and scores on the PHQ-15. (**B**) The significant Spearman correlation for FND-motor (*N*  *=* 39) and PCs (*N*  *=* 55) *between* mean integration values extracted from the cluster shown in red and scores on the PHQ-15. (**C**) The significant Spearman correlation for FND-mixed (*N*  *=* 51) and PCs (*N*  *=* 53) *between* mean segregation values extracted from the cluster shown in red and scores on the PHQ-15. Data points for participants with FND are shown in red, while data points for PCs are shown in blue. Shaded regions reflect 95% confidence intervals. Outliers were removed prior to computing the correlation coefficients if they had a mean weighted-degree, integration or segregation value that exceeded more than 1.5 times the interquartile range above the upper quartile or below the lower quartile. FND-mixed, mixed functional neurological disorder; FND-motor, functional motor disorder; FND-seiz, functional seizures; PCs, psychiatric controls.

In FND-mixed alone (and FND-motor or FND-seiz subtypes), there were no significant correlations with SDQ-20 or PHQ-15 scores. Across FND and PC groups, we observed several significant relationships with functional connectivity metrics and PHQ-15 scores, a measure of patient-reported somatic symptoms. Mean left IFG cluster weighted-degree values correlated with PHQ-15 scores across FND-seiz and PCs (*r_s_*(75) = 0.28, *P*_corrected_ = 0.02; Fig. 5A). Mean integration values extracted from a cluster spanning the left primary sensorimotor areas, posterior insula/posterior STG and bilateral dorsomedial cortices correlated with PHQ-15 scores across FND-motor and PCs (*r_s_*(93) = 0.25, *P*_corrected_ = 0.04; Fig. 5B). Mean dorsal ACC cluster segregation values correlated with PHQ-15 scores across the FND-mixed group and PCs (*r_s_*(105) = 0.30, *P*_corrected_ = 0.005; Fig. 5C), as well as the FND-motor and PCs (*r_s_*(94) = 0.29, *P*_corrected_ = 0.01) and FND-seiz and PCs (*r_s_*(70) = 0.32, *P*_corrected_ = 0.01).

## Discussion

This study characterized the functional network architecture of patients with FND compared to age, sex, depression, anxiety and PTSD severity-matched PCs, while also contextualizing results in relation to age- and sex-matched HCs. Noteworthy findings included the following: (i) *Increased rsFC* weighted-degree of the right dorsal ACC, SFG, left IFG and SMA for FND-mixed and its motor and seizure subtypes versus PCs. Across FND-seiz and PCs, somatic symptom severity (PHQ-15 scores) correlated with increased left IFG weighted-degree. (ii) *Increased between-network integration* connections in somatomotor-associated brain areas for FND-mixed, FND-motor and FND-seizure versus PCs. Heightened connections between the SMN and all six other Yeo *et al.*^51^ resting-state networks were observed (particularly with the DMN, FPN and SN). Across FND-motor and PCs, somatic symptom severity correlated with increased integrated connectivity for several somatomotor and SN-associated regions. (iii) *Increased within-network segregation* connections for the FPN portion of the dorsal ACC for all three FND groups versus PCs. Across FND-mixed and PCs (as well as for FND-motor and FND-seiz versus PCs), increased dorsal ACC segregated connectivity positively correlated with PHQ-15 scores. (iv) The above between-group findings remained significant across primary adjustments for age, sex and SSRI/SNRI use and *post hoc* corrections for depression, anxiety and PTSD severity scores and for childhood maltreatment burden. (v) Individual connectivity values were predominantly within the range of HCs, with FND participants generally having slightly higher values and PCs having slightly lower values, suggesting that alterations in FND likely represent subtle shifts in network architecture. (vi) Additionally, the results highlight some FND subtype differences. Overall, these findings support that FND represents a problem of both abnormal within- and between-network communication, with a pronounced increase in between-network rsFC between the SMN and all other resting-state networks.

Across FND versus PC analyses, increased weighted-degree was observed in the left IFG and right DMPFC, DMN regions previously implicated in FND neurobiology.^22,33,57-59^ For example, increased rsFC within DMN nodes has been previously characterized in patients with functional limb weakness.^60^ Increased IFG activation has also been reported during passive movement in functional weakness, suggested to potentially represent heightened motor inhibition.^61^ Beyond motor inhibition, increased IFG activity has also been observed in individuals with functional versus essential tremor.^57^ In FND-seiz versus HCs, the IFG was shown to have increased nodal-degree (an unweighted centrality metric).^32^ Additionally, in patients with FND-seiz, the IFG showed increased connectivity with the central sulcus, anterior and posterior cingulate cortices, correlating with dissociation severity.^21^ These mechanistically relevant correlations^21^ observed for dissociative symptoms align with our observation of somatic symptom severity scores across FND-seiz and PCs positively correlating with left IFG weighted-degree scores.

In integration analyses, heightened rsFC between nodes of the SMN (including pre- and postcentral gyri and the posterior insula) and other functional networks were found in FND patients compared to PCs. The pre- and postcentral gyri predominantly exhibited increased rsFC to DMN, FPN and dorsal attention networks, while the posterior insula showed increased connectivity to FPN and SN. In segregation analyses, increased FPN within-network connectivity (specifically for the dorsal ACC) was observed across FND versus PCs; individual differences in segregation within the dorsal ACC cluster correlated with somatic symptom severity across FND and PC subjects. Collectively, these findings fit well with observations of biased attentional processing in patients with FND.^58,59,62,63^

Published rsFC studies in FND have also reported increased connectivity between SN regions and motor control areas, including a positive relationship between the strength of these connections and core FND symptom severity.^17-19,21,27^ Our between-group integration findings, and the positive correlation between PHQ-15 scores (measuring somatic symptom severity) and somatomotor/DMPFC integration across FND-motor and PCs, extend this mechanistic evidence. These findings suggest heightened crosstalk between somatomotor areas and regions conventionally thought to be involved in processes including salience detection, attentional reallocation and multimodal integration, which has previously been interpreted as SN regions intruding upon or ‘hijacking’ somatomotor functions.^64,65^ The SN also supports allostasis and interoception, which is notable given that aberrant neural activation in SN areas has been reported during an interoception task across FND-motor and FND-seiz.^66^ Further inquiry is needed to understand the relationship between observed fMRI alterations and viscerosomatic symptoms, which will help refine the interpretation of our findings.

We also observed patterns unique to FND-motor and FND-seiz, which aligns with growing recognition of both shared and distinct mechanisms across subtypes.^1,6^ Specifically, we observed a pattern of increased weighted-degree centrality and isocortical integration for the FND-motor subtype in regions including the left posterior insula and insular-opercular cortex and the bilateral ACC and MCC. In FND-seiz, we observed a unique pattern of isocortical segregation in the right angular gyrus. These regions have been previously shown to have high discriminative validity in classifying patients with mixed FND from HCs.^28^ These subtype-specific connectivity patterns highlight neurobiological heterogeneity within FND, which has implications for understanding the diverse manifestations of the disorder and for developing potential subtype-specific treatments.^67,68^

Although the above-implicated brain regions have conventionally been discussed in terms of discrete neurocognitive functions, it is increasingly recognized that the functional organization of the brain is more complex than singular mappings suggest.^69^ A predictive processing framework offers an alternative view that permits functional heterogeneity, providing an improved understanding of connectivity amongst large-scale brain networks.^70-76^ An emergent literature has implicated aberrant predictive processing in FND and related conditions.^1,77-79^ A predictive processing framework can be broken down into three key components^70^: (i) *prediction signals* that are generatively constructed by the brain based on past experiences, hypothesized to arise within DMN regions; (ii) *prediction error signals* that reflect differences between predicted sensory inputs and incoming data from the sensory surfaces of the body, hypothesized to be weighted by the SN; and (iii) *precision signals* that modulate prediction and prediction error signals, hypothesized to be set by the FPN. Components of the SMN are also thought to be involved in sending and receiving sensory and motor predictions.^70^ Our findings of an overall increase in weighted connections for regions of the SMN and DMN, specifically for between-network integration connections, could be interpreted as an over-weighting of prediction signals. Additionally, the observed hyperconnectivity of the SMN with the SN might be interpreted as a bias towards maladaptive somatomotor predictions that are not properly updated based on incoming sensory inputs. This could imply a potential down-weighting of incoming sensory signals, which stands in contrast to a sensory amplification hypothesis^79^ and aligns with the growing literature on sensory processing difficulties in FND.^80-82^ This interpretation is also supported by the observed increased dorsal ACC functional segregation, which could reflect a breakdown in the modulation of prediction and prediction error signals by the FPN, such that maladaptive predictions are being maintained and not properly updated. An inability to properly encode prediction errors could result in progressively less precise predictions, which may lead to greater energetic demands and accumulating allostatic burden.^83^ This may exacerbate pre-existing allostatic disruptions, or introduce new problems, which could contribute to fatigue, mood disturbances and other symptoms observed in FND. This is particularly noteworthy in the context of the observed positive associations between connectivity metrics and somatic symptom severity across FND and PCs. Prevalent somatic symptoms such as pain and fatigue, which may arise from allostatic disruptions, have been linked to impaired health-related quality of life and suboptimal clinical outcomes in FND populations.^3,84^ Treatment interventions targeting disruptions in the predictive processing stream and resulting allostatic disruptions may therefore prove useful and should be a focus of future research (e.g. ‘bottom-up’ versus ‘top-down’ forms of psychotherapy, neuromodulation, psilocybin, etc.). Researchers should also seek to investigate whether patterns of heightened somatomotor integration normalizes following successful treatment. An increased propensity for physical symptoms in FND and related conditions has also been proposed to arise from altered emotion category construction (e.g. constructing experiences using non-emotional, bodily concepts rather than emotion concepts).^78^ Other treatments may therefore seek to help individuals re-construct experiences using emotional concepts, which may aid in more efficient allostasis. Overall, our findings of altered functional network architecture in FND, and the relationship between these alterations and somatic symptoms, align well with predictive processing frameworks for FND and functional somatic disorders.^62,77,80,85,86^

This study has limitations. Our findings reflect group-level characteristics and may not reflect mechanisms present in any single patient. It is also possible that more than one pattern of rsFC alterations could lead to the same phenotype, i.e. degenerate mechanisms,^69^ and future work using data-driven methods may help to better elucidate biologically informed subtypes. While we consider the reported findings to be likely disorder-related given our contextualization with PCs and controlling for covariates, they may also reflect compensatory mechanisms. Further, regions that did not hold across *post hoc* adjustments, such as the ventromedial prefrontal cortex in integration analyses, might still hold mechanistic relevance. As such, modelling affective symptoms and risk factors as potential causal factors in a complex system, rather than treating them as covariates, may provide additional mechanistic insights.^69^ Additionally, standard functional network parcellations only represent isocortical components,^51^ thereby restricting our integration and segregation analyses to the isocortex. Future work should aim to understand integrated and segregated connectivity in subcortical structures. The specific seven-network parcellation we employed was also relatively coarse.^51^ The use of more fine-grained parcellations may yield different results, especially for segregation analyses, where more granular parcellations could reduce the number of within-network connections and alter findings. Additionally, parcellation-based approaches assume a one-to-one mapping between each brain voxel and a given network, despite growing recognition that brain networks are dynamic and overlapping.^87^ Our analyses also only focused on static rsFC, and recent advances have also revealed dynamic functional connectivity alterations in FND.^24,25^ Lastly, more research is needed to identify relationships between the functional network architecture and core FND symptoms—efforts that likely will be done in the context of developing new outcome measures.^43^

In conclusion, our findings elucidate how intrinsic functional network architecture alterations may contribute to the pathophysiology of FND. Compared to PCs, we observed widespread changes in brain network organization for regions of the SMN, SN, DMN and FPN, offering novel insights into aberrant mechanisms in FND. Using PCs highlights that the observed differences are not merely due to comorbid psychiatric conditions, underscoring their FND specificity. Additionally, the relationships between several connectivity measures and somatic symptom severity across FND and PC groups suggest that the observed neural alterations hold transdiagnostic mechanistic relevance. Future research should seek to validate these findings in larger-scale, multicentre studies.

## Supplementary Material

fcaf195_Supplementary_Data

## Data Availability

For qualified researchers, de-identified data pertaining to study results can be made available upon reasonable request, pending local IRB approval. Analysis code is available at https://github.com/FND-RG-Lab/NetworkIntegrationSegregation.
