# Supplementary material for: Delineating network integration and segregation in the pathophysiology of functional neurological disorder
Source: Brain Commun. 2025 May 21;7(3):fcaf195. doi: 10.1093/braincomms/fcaf195 (PMC12107243; doi:10.1093/braincomms/fcaf195)
Supplement: fcaf195_Supplementary_Data [file fcaf195_supplementary_data.zip › Supplementary_tables_and_legend_for_Supplementary_Figure_1.docx]

**SUPPLEMENTARY MATERIALS**

**MRI Acquisition**

Subjects were scanned on the same Siemens Tim Trio 3T MRI scanner using a 12-channel phased-array head coil. A high-resolution T1-weighted magnetization-prepared rapid gradient echo (MP-RAGE) scan was acquired for each subject with the following parameters: 1mm isotropic voxels; 160 sagittal slices; acquisition matrix size=256x256; repetition time=2300ms; echo time=2.98ms; field of view=256mm. Resting-state blood oxygen level-dependent scans were acquired using T2*-weighted echo-planar imaging sequences with the following parameters: TR=3000ms; TE=30ms; flip angle 85; 216mm FOV; 3mm isotropic voxels; sequence length=6 minutes, 12 seconds (124 time points/scan). All participants with FND, and all but 24 control participants, had two functional acquisitions (4 PCs, 20 HCs had one scan). During scanning, participants were instructed to remain as still as possible with their eyes open. Bi-temporal foam pads restricted head motion, and earplugs were used to attenuate scanner noise.

**Supplementary Fig. 1. Supplementary results from graph theory analyses in functional neurological disorder (FND) vs. matched psychiatric controls (PCs). (A)** Results from whole-brain weighted-degree functional connectivity analyses. *Left panel* depicts intersecting voxels that held across all weighted-degree analyses. Maps were computed as the intersection of binarized maps shown in Fig. 2A, across all primary and post-hoc adjustments. These regions included the left inferior frontal gyrus (IFG) and opercular cortex (cluster 1) and the right anterior cingulate cortex (ACC), superior frontal gyrus (SFG), and bilateral supplementary motor area (SMA) (cluster 2). *Right panel* depicts plots for FND-mixed (*N=*61) and PC (*N=*58) participants showing mean weighted-degree extracted from cluster 1 and 2 as labeled in panel (B); visualized data is from the FND-mixed vs. PCs analysis. **(B)** Results from isocortical integration analyses. *Left panel* depicts intersecting voxels that held across all integration analyses. Maps were computed as the intersection of binarized maps shown in Fig. 2B, across all primary and post-hoc adjustments. These regions included the bilateral SMA, left pre- and postcentral gyrus (cluster 1), right posterior insula and insular-opercular cortex (cluster 2), and right inferior temporal gyrus (ITG) (cluster 3). *Right panel* depicts violin plots for FND-mixed and PC participants showing mean integration extracted from clusters 1, 2, and 3; visualized data is from the FND-mixed vs. PCs analysis. **(C)** Results from isocortical segregation analyses. *Left panel* depicts intersecting voxels that held across all segregation analyses. Maps were computed as the intersection of binarized maps shown in Fig. 2C, across all primary and post-hoc adjustments. The only point of intersection across them was the bilateral dorsal ACC (cluster 1), which fell within the frontoparietal network. *Right panel* depicts violin plots for FND-mixed and PC participants showing mean segregation extracted from cluster 1; visualized data is from the FND-mixed vs. PCs analysis.

**Supplemental Table 1. Demographic and psychometric characteristics of functional motor disorder (FND-motor) and functional seizure (FND-seiz) subtypes.**

|  | **FND-motor** (N = 46)  Mean ± SD or N | **FND-seiz** (N = 23)  Mean ± SD or N |
| --- | --- | --- |
| **Age (years)** | 41.6 ± 13.4 | 37.1 ± 15.8 |
| **Sex** | F: 41; M: 5 | F: 17; M:6 |
| **Illness Duration** | 4.1 ± 5.4 | 4.3 ± 5.0 |
| **SDQ-20** | 35.5 ± 13.1 | 35.1 ± 10.7 |
| **PHQ-15** | 13.4 ± 6.4 | 13.2 ± 5.5 |
| **BDI-II** | 17.0 ± 12.1 | 16.1 ± 12.6 |
| **STAI-Total** | 82.7 ± 22.5 | 83.0 ± 25.3 |
| **PCL-5** | 27.9 ± 19.1 | 24.4 ± 17.9 |
| **CTQ-Abuse** | 32.5 ± 13.2 | 31.8 ± 13.2 |
| **CTQ-Neglect** | 21.3 ± 8.8 | 21.3 ± 9.8 |
| **SSRI/SNRI** | 22 | 12 |

F, Female, M, Male; SDQ-20, Somatoform Dissociation Questionnaire-20; PHQ-15, Patient Health Questionnaire-15; BDI-II, Beck Depression Inventory-II; STAI-Total, Spielberger State-Trait Anxiety Inventory-Total; PCL-5, Post-Traumatic Stress Disorder Checklist for DSM-5; CTQ, Childhood Trauma Questionnaire; SSRI/SNRI, selective serotonin reuptake inhibitor/serotonin norepinephrine reuptake inhibitor use.

**Supplemental Table 2. Demographic characteristics of participants with functional neurological disorder (FND).**

| **FND Subject** | **FND**  **Subtype** | **Phenotypic Description** | **Current SCID-I**  **Diagnoses** | **Past SCID-I Diagnoses** | **Psychotropic Medications** |
| --- | --- | --- | --- | --- | --- |
| 1 | FND-Seiz | documented functional seizures | - | MDE, PTSD | DLX, LTG |
| 2 | FND-Motor | clinically-established functional tics | ANX NOS | - | CTP |
| 3 | FND-Motor | clinically-established functional tremor, functional gait, & functional limb weakness (left hand, right foot) | PTSD, GAD, Eating Disorder | BPAD-II (with MDE) | BUP, BSP, CLP, GBP, LTG |
| 4 | FND-Motor | clinically-established functional tremor, functional jerks, & functional speech | ANX NOS, Somatoform Pain Disorder | PTSD | GBP, LTA |
| 5 | FND-Motor | clinically-established functional limb weakness (legs) | DYS, Somatization Disorder, GAD | MDE | DLX, BUP, SERT |
| 6 | FND-Seiz, FND-Motor | documented functional seizures, clinically-established functional tremor & functional speech | AG, Social Phobia, Somatoform Pain Disorder, Undifferentiated Somatoform Disorder | MDE | CLP, AMT |
| 7 | FND-Seiz, FND-Motor* | clinically-established functional seizures & clinically-established functional tremor | - | PTSD | - |
| 8 | FND-Seiz | documented functional seizures | - | MDE | SERT |
| 9 | FND-Motor* | clinically-established functional gait | ANX NOS, Undifferentiated Somatoform Disorder | DEP NOS, Specific Phobia | - |
| 10 | FND-Motor* | clinically-established functional limb weakness (left arm/leg) | DYS, MDE, GAD, PD+AG, Somatization Disorder, Hypochondriasis | PTSD | - |
| 11 | FND-Seiz, FND-Motor | documented functional seizures; clinically-established functional limb weakness (left leg) | - | MDE, PTSD, PD-AG | LTG, TZD |
| 12 | FND-Motor | clinically-established functional tremor | Somatoform Pain Disorder | ETOH Abuse, ANX NOS | DLX, CLP |
| 13 | FND-Seiz | clinically-established functional seizures | MDE, PD-AG, PTSD | MDE | ECP, QTP |
| 14 | FND-Motor | clinically-established functional limb weakness (arms/legs) | ANX NOS | MDE, PTSD, Eating Disorder | TZD, ECP, BUP |
| 15 | FND-Motor | clinically-established functional jerks, & functional limb weakness (legs) | MDE, PD+AG, PTSD, GAD, Somatoform Pain Disorder | MDE | ECP |
| 16 | FND-Motor | clinically-established functional tremor & functional gait | MDE, SAD | MDE, PD+AG | FLX, CLP |
| 17 | FND-Motor | clinically-established functional tremor & functional gait | DYS, GAD, AG | Eating Disorder | SERT |
| 18 | FND-Seiz | documented functional seizures | PTSD | - | HDZ, ECP, GBP, PZN |
| 19 | FND-Seiz | documented functional seizures | GAD | MDE, PTSD | ECP, DZP |
| 20 | FND-Motor | clinically-established functional gait & functional speech | PTSD, Eating Disorder | MDE | QTP, ECP, CLP, HDZ, PZN |
| 21 | FND-Motor* | clinically-established functional limb weakness (left arm/leg) & functional gait | DYS, PD+AG, PTSD, SSD | MDE, Eating Disorder | LDA, CLP, ECP, QTP, BCP |
| 22 | FND-Seiz | documented functional seizures | DEP NOS | MDE, AG | NRT, LTG |
| 23 | FND-Motor* | clinically-established functional tremor | GAD, IAD | MDE, PTSD | DLX, LRZ |
| 24 | FND-Motor | clinically-established functional jerky movements | GAD | Eating Disorder, AUD, SUMD | ECP, PGB, AMT, APM / DXAM |
| 25 | FND-Motor | functional tremor | ANX NOS | - | GBP |
| 26 | FND-Motor | clinically-established functional jerky movements | GAD | DEP NOS | SERT |
| 27 | FND-Seiz | documented functional seizures | - | DEP NOS, SAD, ANX NOS | LRZ |
| 28 | FND-Seiz | documented functional seizures | PTSD, GAD | MDE | BSP |
| 29 | FND-Motor | clinically-established functional limb weakness (left arm) & functional speech | GAD, SSD | MDE, PTSD | CBD |
| 30 | FND-Seiz | probable functional seizures | ANX NOS | PTSD, GAD, MDE | SERT, TZD, PZN |
| 31 | FND-Seiz | probable functional seizures | - | ANX NOS | - |
| 32 | FND-Seiz, FND-Motor | clinically-established functional tremor, probable functional seizures | - | MDE | AMT |
| 33 | FND-Seiz, FND-Motor | documented functional seizures, clinically-established functional limb weakness (left leg) & functional gait | MDE, GAD, PD+AG | MDE | CLP, FLX, PZN |
| 34 | FND-Seiz | documented functional seizures | GAD | DEP NOS, AUD | FLX, APZ |
| 35 | FND-Motor* | clinically-established functional tremor & functional speech | PTSD, GAD, PD+AG, MDE | - | DLX, BUP, LRZ |
| 36 | FND-Motor | clinically-established functional gait & functional speech | AG, ADHD | - | APZ, CBD |
| 37 | FND-Seiz, FND-Motor | documented functional seizures; clinically-established functional gait & functional speech | ANX NOS | PTSD, PD+AG | PGB, CBD |
| 38 | FND-Motor | clinically-established functional gait, functional tremor, functional limb weakness (right arm/leg), & functional speech | GAD, PD+AG | PTSD, MDE | TPM |
| 39 | FND-Motor* | clinically-established functional gait | ANX NOS, SSD | GAD, MDE | SERT, LRZ, GBP |
| 40 | FND-Motor* | clinically-established functional gait & functional limb weakness (right arm/leg) | - | MDE, PTSD, Eating Disorder | GBP, AMP, TZD, PZN |
| 41 | FND-Motor | clinically-established functional tremor | - | PTSD, BPAD-II (with MDE) | LTM, QTP, LRZ |
| 42 | FND-Motor | clinically-established functional tremor, functional dystonia & functional gait | PD+AG, SSD | GAD, DEP NOS | - |
| 43 | FND-Motor | clinically-established functional limb weakness (legs) & functional speech | - | DEP NOS, Specific Phobia | - |
| 44 | FND-Motor | clinically-established functional facial spasms/tics & functional speech | GAD, PD+AG, ADHD, PTSD | MDE | CLN, LDA, APR, SERT, LRZ |
| 45 | FND-Motor | clinically-established functional gait & functional speech | - | DEP NOS, ANX NOS | GBP |
| 46 | FND-Motor | clinically-established functional limb weakness (left leg) | ADHD | DYS, MDE, GAD, SAD | DLX, LDA, PGB |
| 47 | FND-Motor | clinically-established functional jerks/spasms/tics | PTSD, ADHD | SAD, MDE, Specific Phobia | MIR, SERT, MPD, LRZ |
| 48 | FND-Seiz | documented functional seizures | GAD, ADHD | MDE, AG | LTG, NRT, FLX, MPD |
| 49 | FND-Motor | clinically-established functional limb weakness (right arm/leg) & functional speech | PTSD, GAD, MDE, PD+AG, SSD | MDE | APR, FLX, GBP, LRZ, MLT |
| 50 | FND-Motor | clinically-established functional tremor & functional dystonia (right foot) | MDE, GAD, PTSD | MDE, PD+AG, SAD | BSP |
| 51 | FND-Seiz | documented functional seizures | GAD, PTSD, ADHD | Eating Disorder, MDE, PD+AG | OLZ, CTP, APM / DXAM |
| 52 | FND-Seiz | documented functional seizures | MDE, GAD | MDE, SAD, Eating Disorder | LTG, LRZ |
| 53 | FND-Motor | clinically-established functional limb weakness (left leg) | ANX NOS | MDE, ANX NOS | GBP, HDZ, ECP |
| 54 | FND-Seiz, FND-Motor | probable functional seizures; clinically-established functional limb weakness (bilateral leg), functional tremor, & functional gait | SSD, AG, DYS | MDE, PD-AG, GAD, PTSD, Eating Disorder | AMT, DLX |
| 55 | FND-Motor | clinically-established functional dystonia & functional speech | GAD | PTSD | - |
| 56 | FND-Motor | clinically-established functional limb weakness (left arm & leg), functional tremor, & functional jerks | - | PTSD | - |
| 57 | FND-Motor | clinically-established functional tremor | PTSD, MDE Anxiety NOS | - | MIR, CLP |
| 58 | FND-Motor | clinically-established functional tremor* | PTSD, MDE, SAD, GAD, SSD | DYS, PTSD, AUD, ANX NOS | APZ, BSP; PZN, ECP, TZD, HDZ |
| 59 | FND-Seiz, FND-Motor | documented functional seizures; clinically-established* functional jerks/tics, & functional speech | PD+AG, DYS, GAD, SSD | OCD | DLX, GBP, LRZ |
| 60 | FND-Seiz | documented functional seizures | AG, SAD, OCD, SSD | - | HDZ, DZP |
| 61 | FND-Motor | clinically-established functional tremor, & functional gait | Anxiety NOS | - | HDZ |

*Indicates subject also had concurrent functional somatosensory loss (e.g., non-dermatomal somatosensory deficits). Subjects 1-17 were evaluated using a SCID-I for DSM-IV-TR, while subjects 18-61 were evaluated using the SCID-I for DSM-5; subject 18 had missing SCID-I data and psychiatric comorbidities are based on chart-review diagnoses. M, Male; F, Female; FND-Motor, Functional Motor Disorder; FND-Seiz, Functional Seizures; ADHD, Attention-Deficit/Hyperactivity Disorder; AG, Agoraphobia; ANX, Anxiety; AUD, Alcohol Use Disorder; BPAD, Bipolar Affective Disorder; DEP, Depression; DYS, Dysthymia; ETOH-Abuse, Alcohol Abuse; GAD, Generalized Anxiety Disorder; IAD, Illness Anxiety Disorder; MDE, Major Depressive Episode; NOS, not otherwise specified; OCD, Obsessive Compulsive Disorder; PD+AG, Panic Disorder with Agoraphobia; PD-AG, Panic Disorder without Agoraphobia; PTSD, Post-Traumatic Stress Disorder; SAD, Social Anxiety Disorder; SSD, Somatic Symptom Disorder; SUD, Substance Use Disorder; SUMD, Substance Use Related Mood Disorder; AMT, Amitriptyline; APM, Amphetamine; APR, Aripiprazole; APZ, Alprazolam; BCP, Baclophen; BSP, Buspirone; BUP, Bupropion; CBD, Cannabidiol; CLN, Clonidine; CLP, Clonazepam; CTP, Citalopram; DLX, Duloxetine; DXAM, Dextroamphetamine; DZP, Diazepam; ECP, Escitalopram; FLX, Fluoxetine; GBP, Gabapentin; HDZ, Hydroxyzine; LDA, Lisdexamfetamine; LTM, Lithium; LTG, Lamotrigine; LRZ, Lorazepam; LTA, Levetiracetam; MIR, Mirtazapine; MLT, Melatonin; MPD, Methylphenidate; NRT, Nortriptyline; OLZ, Olanzapine; PGB, Pregabalin; PZN, Prazosin; QTP, Quetiapine; SERT, Sertraline; TPM, Topiramate; TZD, Trazodone.

**Supplemental Table 3. Demographic characteristics of psychiatric controls (PC) with a lifetime history of clinically-salient depression, anxiety and/or post-traumatic stress disorder.**

| **PC**  **Subject** | **Current SCID-I**  **Diagnoses** | **Past SCID-I**  **Diagnoses** | **Psychotropic**  **Medications** |
| --- | --- | --- | --- |
| 1 | - | MDE, PTSD | - |
| 2 | - | DEP NOS | - |
| 3 | - | PTSD | - |
| 4 | - | DEP NOS | - |
| 5 | - | ANX NOS | - |
| 6 | GAD | MDE, PD+AG, Eating Disorder | CTP |
| 7 | DEP NOS | PTSD, MDE | SERT, LTG, TZD, QTP |
| 8 | ANX NOS | MDE | BUP, SERT |
| 9 | GAD, MDE, Eating Disorder | PD-AG, PTSD | BUP, LTG, LRZ, LTM, TZD |
| 10 | MDE | Specific Phobia | - |
| 11 | - | PTSD, DEP NOS | CTP |
| 12 | DYS, ANX NOS | MDE, PD+AG | BUP, SERT |
| 13 | MDE, Eating Disorder, PTSD, GAD | - | DLX |
| 14 | ANX NOS | ANX NOS, DEP NOS, Eating Disorder, ETOH Abuse | VEN |
| 15 | BPAD-II (current mild depression) | MDE | DLX, LTG, QTP |
| 16 | DEP NOS, PD+AG, PTSD, Social Phobia | MDE | DLX, CLP, LTG, QTP |
| 17 | MDE | MDE, ANX NOS | BUP, DLX |
| 18 | MDE, PTSD | MDE, AUD | ZPD, MPD, TZD, MIR, GBP, TPM |
| 19 | GAD, DEP NOS | MDE, PD-AG | ECP |
| 20 | MDE, GAD | PTSD | BUP, LTG, BSP |
| 21 | GAD | MDE, Eating Disorder, Anxiety NOS | FLX, LRZ |
| 22 | MDE, GAD | PTSD | FLX, MIR |
| 23 | MDE, DYS, GAD, AG | - | ECP, APR |
| 24 | - | MDE, ANX NOS | - |
| 25 | ANX NOS | MDE | - |
| 26 | DEP NOS, ANX NOS | MDE | SERT, QTP, GBP |
| 27 | GAD | MDE | BUP, MLT |
| 28 | DYS, ANX NOS | MDE, AUD | BSP, SERT |
| 29 | GAD, SAD | - | SERT |
| 30 | MDE, PTSD, GAD, OCD | - | BSP, LTG, BUP, APM / DXAM, TZD, MLT |
| 31 | Eating Disorder | MDE, PD-AG, OCD | - |
| 32 | MDE, GAD, Specific Anxiety | MDE, PTSD, OCD, Eating Disorder | SERT, LTG, ECP, TZD, MLT |
| 33 | PTSD, SAD, Eating Disorder, ADHD | MDE | TPM, APM / DXAM |
| 34 | MDE, ANX NOS | MDE | LTG, TZD, LRZ |
| 35 | ANX NOS | DEP NOS, GAD | SERT |
| 36 | GAD | - | ECP, BUP, APZ |
| 37 | - | GAD, DEP NOS | BUP |
| 38 | - | ANX NOS | SERT |
| 39 | - | MDE | - |
| 40 | - | MDE, Specific Phobia | - |
| 41 | DEP NOS, ANX NOS | MDE | DLX, BUP |
| 42 | - | MDE, PD+AG | - |
| 43 | - | DEP NOS | - |
| 44 | - | PTSD, MDE | - |
| 45 | GAD | MDE | - |
| 46 | - | DEP NOS | - |
| 47 | - | MDE, PTSD, PD+AG, OCD, AUD | - |
| 48 | PTSD, SAD, ADHD, PD+AG | MDE, Eating Disorder | LDA |
| 49 | - | PTSD | - |
| 50 | - | MDE | GBP, CTP |
| 51 | GAD, MDE, SAD, Specific Phobia | DYS, PTSD | CLP |
| 52 | PTSD | MDE | ECP, CLN |
| 53 | Specific Phobia | - | LRZ |
| 54 | GAD, MDE | - | ECP |
| 55 | MDE, Anxiety NOS, MDE | GAD, MDE, AUD, PD+AG | - |
| 56 | - | MDE | - |
| 57 | PTSD, GAD, MDE, DYS | Eating Disorder | VEN, MPD, LRZ, PZN, QTP, GBP, BPN, EZP |
| 58 | - | OCD, GAD | ECP |

Subjects 1-20 were evaluated using a SCID-I for DSM-IV-TR, while subjects 20-58 were evaluated using the SCID-I for DSM-5. Clinically-salient depression was defined as any subject with diagnoses of BPAD with MDE, DEP NOS, DYS, and/or MDE. Clinically-salient anxiety was defined as AG, ANX NOS, GAD, OCD, PD ± AG, SAD, Specific Phobia, and/or Social Phobia. M, Male; F, Female; ADHD, Attention-Deficit/Hyperactivity Disorder; AG, Agoraphobia; ANX, Anxiety; AUD, Alcohol Use Disorder; BPAD, Bipolar Affective Disorder; DEP, Depression; DYS, Dysthymia; ETOH-Abuse, Alcohol Abuse; GAD, Generalized Anxiety Disorder; MDE, Major Depressive Episode; NOS, not otherwise specified; OCD, Obsessive Compulsive Disorder; PD+AG, Panic Disorder with Agoraphobia; PD-AG, Panic Disorder without Agoraphobia; PTSD, Post-Traumatic Stress Disorder; SAD, Social Anxiety Disorder; APM / DXAM, Amphetamine / Dextroamphetamine; APR, Aripiprazole; APZ, Alprazolam; BPN, Buprenorphine-naloxone; BSP, Buspirone; BUP, Bupropion; CLN, Clonidine; CLP, Clonazepam; CTP, Citalopram; DLX, Duloxetine; ECP, Escitalopram; EZP, Eszopiclone; FLX, Fluoxetine; GBP, Gabapentin; LDA, Lisdexamfetamine; LRZ, Lorazepam; LTG, Lamotrigine; LTM, Lithium; MIR, Mirtazapine; MLT, Melatonin; MPD, Methylphenidate; PZN, Prazosin; QTP, Quetiapine; SERT, Sertraline; TPM, Topiramate; TZD, Trazodone; VEN, Venlafaxine; ZPD, Ziprasidone
