# Supplementary figures and images for: Delineating network integration and segregation in the pathophysiology of functional neurological disorder
Source: Brain Commun. 2025 May 21;7(3):fcaf195. doi: 10.1093/braincomms/fcaf195 (PMC12107243; doi:10.1093/braincomms/fcaf195)

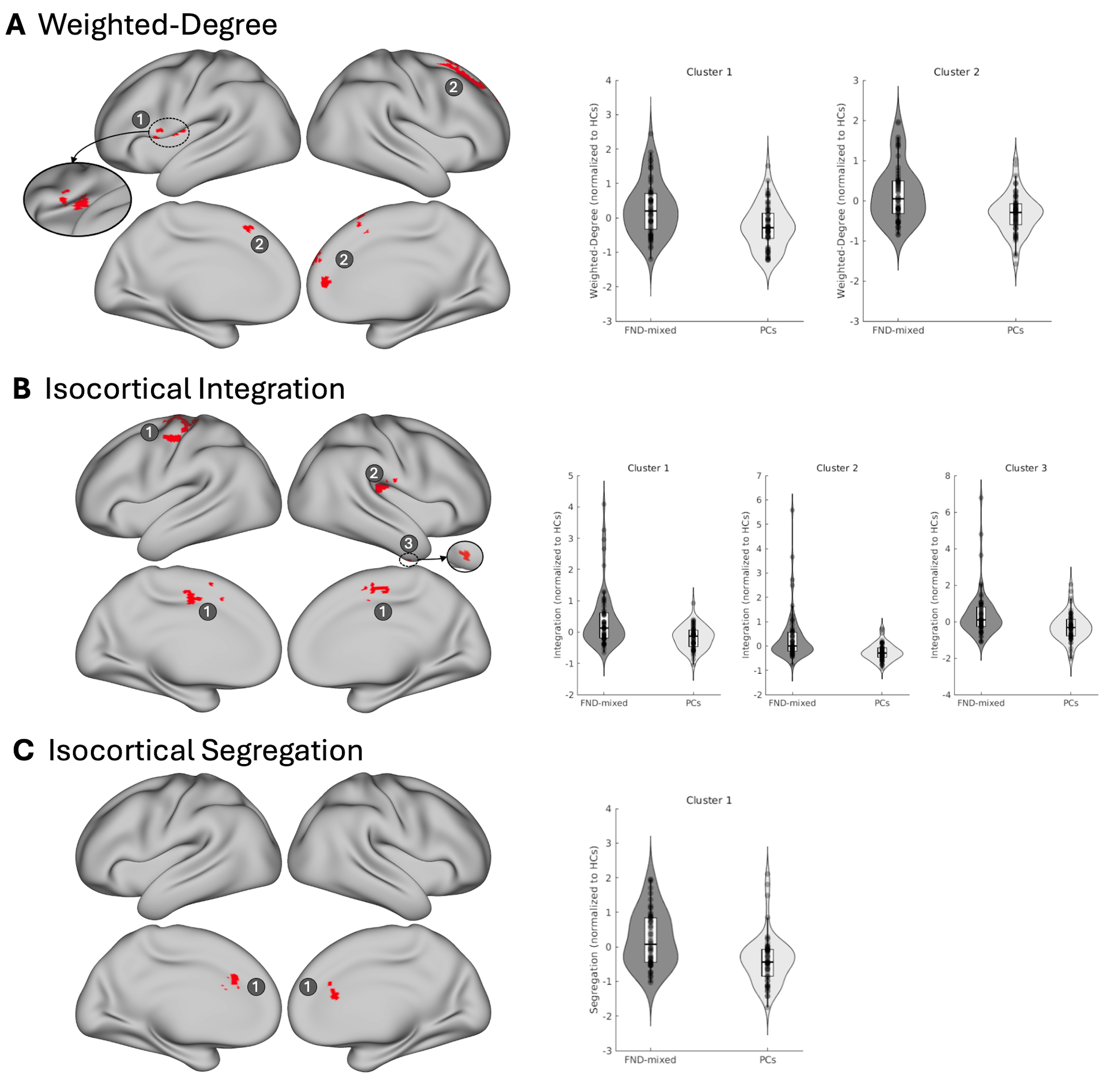

Supplement: fcaf195_Supplementary_Data [file fcaf195_supplementary_data.zip › Supplementary_Figure_1.tif]
